# Complete Genome Sequences of Two *Pseudomonas aeruginosa* Strains Isolated from Children with Bacteremia

**DOI:** 10.1128/genomeA.00927-17

**Published:** 2017-08-31

**Authors:** Luis F. Espinosa-Camacho, Gabriela Delgado, Guadalupe Miranda-Novales, Gloria Soberón-Chávez, Luis D. Alcaraz, Rosario Morales-Espinosa

**Affiliations:** a Departamento de Microbiología y Parasitología, Facultad de Medicina, Universidad Nacional Autónoma de México, Mexico City, Mexico; b Unidad de Investigación en Epidemiología Hospitalaria, Centro Médico Nacional Siglo XXI, Mexico City, Mexico; c Departamento de Biología Molecular y Biotecnología, Instituto de Investigaciones Biomédicas, Universidad Nacional Autónoma de México, Mexico City, Mexico; d Laboratorio Nacional de Ciencias de la Sostenibilidad, Instituto de Ecología, Universidad Nacional Autónoma de México, Mexico City, Mexico

## Abstract

Two *Pseudomonas aeruginosa* strains isolated from children with bacteremia in Mexico City were sequenced using PacBio RS-II single-molecule real-time (SMRT) technology. The strains consist of a 7.0- to 7.4-Mb chromosome, with a high content of mobile elements, and variation in the genetic content of class 1 integron In1409.

## GENOME ANNOUNCEMENT

*Pseudomonas aeruginosa* is associated with chronic recurrent pulmonary infections that are responsible for high mortality in children with underlying conditions such as hematology-oncology diseases, extended hospitalization in the intensive care unit (ICU), and prematurity (1).

Two *P. aeruginosa* strains (Pa1207 and Pa1242) were sequenced. The strains were isolated from children with bacteremia admitted to a pediatric hospital in Mexico City. Strain Pa1207 was resistant to different β-lactams (including carbapenems and cephalosporins), amikacin, and tobramycin but was susceptible to gentamicin, polymyxin B, and fluoroquinolones. Strain Pa1242 presented only intermediate resistance to polymyxin B and was susceptible to 19 antimicrobials tested.

The genomic DNA of the strains was purified with the DNeasy blood and tissue kit (Qiagen) and sent to the Yale Center for Genome Analysis for PacBio RS II single-molecule real-time (SMRT) sequencing. A standard library of 20-kb fragments was prepared and sequenced on two SMRT cells with P4-C2 chemistry. The continuous long reads were assembled using the HGAP/Quiver protocol in SMART Portal v3 (2). The final assemblies had mean coverages of ∼146× and ∼181× for Pa1207 and Pa1242, respectively, and consisted of chromosomes of 7,411,863 bp and 7,050,510 bp, with mean G+C contents of 65.7% and 65.8%, respectively. A total of 7,153 genes were annotated for Pa1207: 7,072 CDSs, 65 tRNAs, 12 rRNAs, 4 noncoding RNAs (ncRNAs), and 247 pseudogenes. For strain Pa1242, 6,735 genes were annotated: 6,654 CDSs, 65 tRNAs, 12 rRNAs, 4 ncRNAs, and 346 pseudogenes.

The sequences were annotated using the NCBI Prokaryotic Genome Annotation Pipeline (http://www.ncbi.nlm.nih.gov). The annotation was manually curated and enriched by the presence of mobile elements, antibiotic resistances genes, efflux pumps, and potential virulence factors using the IslandPath-DIMOB, SIGI-HMM, IslandPick (3), CARD (4), VirulenceFinder (5), and Integrall (6) databases. Strain Pa1207 presented 4 genomic islands, 10 prophages, and 3 integrative plasmids. Strain Pa1242 presented 4 different genomic islands and 8 prophages.

In both strains, there are three large genomic islands inserted at previously identified loci (7–10) but with different genetic compositions. One island conserved the first 31 open reading frames (ORFs) described in *P. aeruginosa* genome island 1 (PAGI-1), and the other had only the 4 ORFs described in pathogenicity island 2 (PAPI-2). Strain Pa1207 presents a mobile element shared with *Pseudomonas fluorescens*, which encodes to dehydrogenases, type VI secretion system components, and hypothetical proteins. Other islands in both strains are hybrids formed for genes from PAPI-1/pKLC-102, where a group of genes of the major pilins are conserved.

The two strains presented efflux systems MexAB-OprM, MexCD-OprJ, MexEF-OprM, and MexXY, the porin OprD, and the β-lactam OXA-50. Strain Pa1207 presents an integron class 1 (In1409) not previously described (http://integrall.bio.ua.pt/?acc=CP022001). The integron carried genes *AAC*(*6′*)-*33*, *aadA6*, *bla*OXA-2, and *sul1*. The two strains presented type III secretion system (*exoU*, *exoS* and *exoT, exoY*) genes.

### Accession number(s).

These whole-genome projects have been deposited in GenBank under the nucleotide accession no. CP022001 (Pa1207) and CP022002 (Pa1242), BioSample no. SAMN05020325 (Pa1207) and SAMN05020326 (Pa1242), and BioProject no. PRJNA389181.
